# Elevated Cortisol Levels and Manic Symptoms in a 16-Year-Old Female: A Case Report

**DOI:** 10.7759/cureus.61693

**Published:** 2024-06-04

**Authors:** Ateeba Ahmed, Pradeep S Patil

**Affiliations:** 1 Psychiatry, Jawaharlal Nehru Medical College, Datta Meghe Institute of Higher Education and Research, Wardha, IND

**Keywords:** psychiatric evaluation, adolescents, cortisol, bipolar disorder, mania

## Abstract

This case report presents the clinical presentation, diagnosis, and management of a 16-year-old female with elevated cortisol levels who was diagnosed with mania. The patient exhibited symptoms consistent with a manic episode, including extreme euphoria, decreased need for sleep, impulsivity, and heightened irritability. Laboratory investigations revealed an elevated morning cortisol level, prompting further psychiatric evaluation. A diagnosis of bipolar I disorder, manic episode, was made based on established criteria. The patient was initiated on mood stabilizers and antipsychotic medications alongside psychoeducation for the patient and her family. This case underscores the potential association between cortisol dysregulation and mood disorders, highlighting the importance of comprehensive assessment and personalized treatment approaches in adolescents with bipolar disorder. Further research is needed to elucidate the underlying mechanisms linking cortisol dysregulation and mood disturbances and explore novel therapeutic interventions targeting hypothalamic-pituitary-adrenal axis dysfunction.

## Introduction

Mania, characterized by elevated mood, increased energy, and impaired judgment, is a hallmark feature of bipolar disorder [1]. The etiology of bipolar disorder is complex and multifactorial, involving genetic, neurobiological, and environmental factors. Hormonal dysregulation, particularly alterations in the hypothalamic-pituitary-adrenal (HPA) axis and elevated cortisol levels, has garnered attention for its potential role in the pathogenesis of mood disorders, including bipolar disorder [2]. Cortisol, the primary glucocorticoid hormone secreted by the adrenal glands in response to stress, is crucial in regulating various physiological processes, including metabolism, immune function, and the stress response. Dysregulation of the HPA axis and aberrant cortisol secretion have been implicated in the pathophysiology of mood disorders such as depression and bipolar disorder [3].

Studies have demonstrated alterations in cortisol secretion patterns and increased cortisol levels during manic and depressive episodes in individuals with bipolar disorder [4]. Furthermore, elevated cortisol levels have been associated with symptom severity, functional impairment, and poor treatment response in patients with bipolar disorder [5]. The relationship between cortisol dysregulation and mood disorders is bidirectional, with stress serving as a precipitating factor for mood episodes and mood disturbances affecting HPA axis function and cortisol secretion [6]. Neurobiological mechanisms underlying this association include alterations in neurotransmitter systems, dysregulation of glucocorticoid receptor signaling, and structural changes in brain regions implicated in mood regulation [7].

Despite the growing body of evidence implicating cortisol dysregulation in the pathophysiology of mood disorders, including bipolar disorder, the precise mechanisms underlying this relationship remain incompletely understood. Further research is needed to elucidate the complex interplay between hormonal, neurobiological, and environmental factors in the development and progression of mood disorders to identify novel therapeutic targets and interventions.

## Case presentation

A 16-year-old Caucasian female presented to our psychiatric clinic with a referral from her family physician due to concerns regarding her increasingly erratic behavior and mood swings over the past few months. The patient, previously described as outgoing and academically successful, had been experiencing periods of extreme euphoria, decreased need for sleep, impulsivity, and heightened irritability. She reported racing thoughts, grandiose ideas, and increased goal-directed activities, including reckless spending and impulsive decision-making.

Upon examination, the patient exhibited pressured speech, increased psychomotor activity, and a euphoric mood. Physical examination revealed no significant abnormalities. Laboratory investigations noted an elevated morning cortisol level of 28 μg/dL (normal range: 5-23 μg/dL). Other laboratory tests were within normal limits, including a complete blood count, comprehensive metabolic panel, thyroid function tests, and urine drug screen. The patient denied any significant medical or psychiatric history, including substance use. Family history was notable for bipolar disorder in her maternal aunt. Given the patient's symptoms of mania and the presence of elevated cortisol levels, further psychiatric evaluation was pursued. A diagnosis of bipolar I disorder, manic episode, was made according to the criteria outlined in the Diagnostic and Statistical Manual of Mental Disorders, Fifth Edition (DSM-5) [8].

The patient was started on divalproex sodium and quetiapine for mood stabilization and symptom management. Psychoeducation regarding bipolar disorder and the importance of medication compliance was provided to the patient and her family. Regularly monitoring symptoms, adverse effects, and therapeutic response were initiated with scheduled follow-up appointments.

## Discussion

The case presented herein underscores the potential association between elevated cortisol levels and the manifestation of manic symptoms in adolescents with bipolar disorder. Cortisol, a glucocorticoid hormone secreted by the adrenal glands in response to stress, is crucial in regulating various physiological processes, including metabolism, immune response, and mood regulation. Dysregulation of the HPA axis, leading to aberrant cortisol secretion, has been implicated in the pathophysiology of mood disorders, including bipolar disorder [9]. Several mechanisms may underlie the relationship between cortisol dysregulation and the development of manic symptoms. Firstly, cortisol interacts with various neurotransmitter systems, including serotonin, dopamine, and glutamate, which are implicated in the pathogenesis of mood disorders. Dysregulated cortisol levels may alter the balance of these neurotransmitters, contributing to mood dysregulation and the onset of manic episodes [2,10]. Secondly, cortisol exerts feedback control on the HPA axis via glucocorticoid receptors in the hypothalamus and pituitary gland. Dysregulation of this feedback mechanism may lead to excessive cortisol secretion and subsequent mood disturbances [11].

The findings of elevated cortisol levels in our patient align with previous studies reporting similar observations in individuals with bipolar disorder, particularly during manic episodes [12,13]. Furthermore, genetic factors may contribute to the interplay between cortisol dysregulation and mood disorders. Family studies have shown a heritable component to both bipolar disorder and alterations in the HPA axis function, implicating shared genetic vulnerabilities [14,15]. In our case, the patient's family history of bipolar disorder further supports the potential genetic predisposition to cortisol dysregulation and mood disturbances. The management of bipolar disorder with comorbid cortisol dysregulation poses unique challenges. While mood stabilizers and antipsychotic medications are commonly used to manage manic symptoms, adjunctive interventions targeting cortisol regulation may be beneficial. For instance, psychotherapeutic approaches, such as cognitive-behavioral therapy and mindfulness-based interventions, have shown promise in modulating HPA axis activity and reducing cortisol levels in individuals with mood disorders [14,15].

## Conclusions

This case report highlights the potential association between elevated cortisol levels and the manifestation of manic symptoms in adolescents with bipolar disorder. The findings underscore the importance of considering hormonal dysregulation as a contributing factor to mood disturbances, particularly in the context of psychiatric evaluation and management. By elucidating the complex interplay between cortisol dysregulation and mood disorders, clinicians can optimize diagnostic accuracy and tailor treatment interventions to address underlying pathophysiological mechanisms. Further research is warranted to understand the neurobiological underpinnings of this relationship better and to develop targeted therapeutic strategies aimed at modulating cortisol levels in individuals with bipolar disorder. A comprehensive, multidisciplinary approach integrating psychiatric, endocrinological, and genetic assessments is essential for achieving optimal outcomes and improving the quality of life for adolescents affected by bipolar disorder and related mood disturbances.
